# The Registration of Deaths Bill

**Published:** 1908-08-08

**Authors:** 


### The Registration of Deaths Bill.

On July 20 the House of Commons ordered to be printed a Bill to amend the law relating to the registration of deaths and to burials, which was presented by Mr. Greenwood and supported amongst others by Sir Walter Foster, Dr. Rutherford, and Dr. Shipman. The object of this bill is, as declared on the cover, to give effect to the principal recommendations put forward by the Select Committee on Death Certification which sat in 1893, at which time, it will be remembered, Sir Walter Foster was Parliamentary Secretary to the Local Government Board. It embodied sundry reforms which are and have for a long time been imperative, though whether there is a better chance of securing their adoption now than when Sir Walter was himself a member of the Government we do not presume to say. For instance, it is enacted that any stillborn child which has issued forth from its mother after the expiration of the twenty-eighth week of pregnancy shall be deemed to have been born alive, and to have died after birth, a curious legal fiction which at least brings the registration of deaths into line with that of births under last year's Act. Further, the certificate of death is to be so framed that any person signing it must have inspected the body, thus abolishing the "as I am informed" clause of the present certificate. The certificate must also specify the signs from which the fact of death is inferred, the cause of death, and, in the case of practising medical men, that the certifier has personally attended the deceased on two occasions, one of these two being within eight days of death. These changes are all salutary of themselves, and may also tend to reassure that section of the public which is much disturbed by contemplating the possibility of premature burial, an occurrence so rare in this country that no case of it has ever been definitely proved.

Other provisions are that every board of guardians shall appoint a public certifier of deaths for each sub-district in their union, who shall be a registered medical practitioner. It is a little difficult to see why the guardians should have control of these officials, especially as the present system of the Poor Law administration is likely to be much modified by the Royal Commission which now has it under discussion; still, that may be allowed to pass for the present. The functions of this officer are as follows: He is to verify and countersign the death certificate given by a coroner after an inquest, and to assist him in this the latter must notify him of the time and place of every inquest, which he must then attend. He is thus, as it were, a medical assessor or adviser to the coroner, an office which would be unnecessary were every coroner a medical man, and which may conceivably produce considerable friction; for there is no machinery provided by which he can compel the coroner to accept his views, and yet he can prevent the burial indefinitely if he declines to confirm the verdict. He is also, when informed by a registrar that there is doubt about any death or about its certification, to inspect the dead body and issue a certificate of death if he thinks fit. This provision is one which distinctly trespasses on ground hitherto allotted to the coroner, for presumably inspection may include that which is usually expressed by the nearly equivalent Greek word autopsy. Thus the public certifier would seem to be given discretionary powers as to the necessity or otherwise of an inquest, a change which, however beneficial on many grounds, has drawbacks on others, and is certain to be resisted. For each death certificate signed by a practitioner, who must himself forward it to the registrar within five days of death, a fee of half a crown is to be paid him; and the same sum is also due should he notify the registrar and coroner of a violent or unnatural death, or of one on which an inquest is desirable.

